# Lipid Complications after Hematopoietic Stem Cell Transplantation (HSCT) in Pediatric Patients

**DOI:** 10.3390/nu12092500

**Published:** 2020-08-19

**Authors:** Gabriela Bis, Wojciech Szlasa, Katarzyna Sondaj, Iga Zendran, Monika Mielcarek-Siedziuk, Ewa Barg

**Affiliations:** 1 Faculty of Medicine, Wroclaw Medical University, 50-367 Wroclaw, Poland; gabrielabis94@gmail.com (G.B.); wojtek.szlasa@outlook.com (W.S.); sondaj.katarzyna@gmail.com (K.S.); igazendran@gmail.com (I.Z.); 2 Department and Clinic of Pediatric Oncology, Haematology and Bone Marrow Transplantation, Wroclaw Medical University, 50-556 Wroclaw, Poland; monika.mielcarek@umed.wroc.pl; 3 Department of Basic Medical Sciences, Wroclaw Medical University, 50-556 Wroclaw, Poland

**Keywords:** HSCT, lipid disorders, thyroid hormones, pediatric patients

## Abstract

HSCT (hematopoietic stem cell transplantation) is a widely applied method of treatment of pediatric patients with leukemia and other bone marrow-associated disorders. Metabolic disturbances can appear as procedure side effects. This study aimed to report incidence of lipid and thyroid disorders and time of their onset in pediatric patients after HSCT. There were 198 pediatric patients (123 males) aged 0.5–20 years who were subjected to HSCT. Patients were mostly diagnosed with Acute Leukemia (*n* = 190). The analysis of lipids, thyroid hormones, and thyroid antibodies levels comprised one month before the HSCT to last follow up visit between 2016 and 2019 (median 3.8 ± 1.8 years after HSCT). In males, the triglycerides levels increased over two times in the course of HSCT in both patients with initially low and elevated HDL (high-density lipoprotein) levels. Most of the lipid disorders occurred in six months after HSCT. Patients treated with L-thyroxine exhibited decreased LDL (low-density lipoprotein) levels. HDL remained at a lower level in males. Thyroid hormone abnormalities were evenly distributed in time until 4 years after HSCT. Patients require long term follow up including lipid metabolism and thyroid function analysis. HSCT survivors demand introduction of polyunsaturated fatty acids into the diet to reduce risk of developing the lipid complications.

## 1. Introduction

Hematopoietic stem cell transplantation (HSCT) is currently a widely used element of treatment programs of malignant disorders [1]. The method is a potentially curative therapeutic approach and is mostly used among patients with hematologic malignancies and myelodysplastic syndromes. It is also introduced in the solid tumors treatment schemes, as well as bone marrow failure syndromes, primary immunodeficiencies, and inborn errors of metabolism [2,3,4]. Depending on the treatment protocol, a patient can be a self-donor (autologous transplantation) or another unrelated person can be a donor for the patient (allogeneic transplantation) [1,2,3]. The procedure involves hematopoietic stem cell collection from peripheral blood after mobilization of stem cells with granulocyte-colony-stimulating factor from bone marrow, bone marrow collection, or cord blood collection from the donor. The allogeneic donor can be related (sibling, parent) or unrelated. A crucial part of HSCT procedure is the conditioning regimen administered before graft infusion. In case of malignant indication, preparative transplant regimens relied on high-dose chemotherapy (agents with nonoverlapping toxicities) or TBI (total body irradiation with supralethal fractionated doses), that ablate marrow hematopoiesis preventing from autologous hematologic recovery, provide sufficient immunoablation to prevent graft rejection and clear residual malignant disease. Depending on indication or clinical condition of a patient conditioning, schemes can be changed to be less intensive. As intensity of conditioning is lowered, toxicities associated with the transplant procedure should be less pronounced [5]. It is not always possible to distinguish which component of the conditioning regimen is responsible for any given toxicity. TBI in childhood can lead to permanent pituitary dysfunction. Growth hormone deficiency, puberty disorders, and central hypothyroidism may appear after many years [6]. High-dose chemotherapy-based regimens consist of many agents, but alkylating chemotherapeutics remain the mainstay of such regimens. A combination of chemotherapeutics can impair metabolism and cause hormonal disparity in HSCT recipients. 

During the allogeneic HSCT procedure, the patients also undergo the immunosuppression process to decrease the chances of HSCT rejection and graft-versus-host disease (GvHD) [7]. The standard immunosuppressive agent is Cyclosporin-A (CSA). Negative CSA influence on lipid profile in organ transplant recipients is well known [8,9]. There are no data regarding pediatric HSCT recipients. In many cases, GvHD may develop anyway [10]. In this situation the drug of first choice is high-dose methylprednisolone. Before transplantation, glucocorticoids are widely used in frontline chemotherapy protocols [11]. Therefore, HSCT recipient’s metabolism and endocrine balance is seriously strained before, during, and after HSCT. Firstly, the patient remains immunocompromised and therefore should be isolated from the environment, which includes restricted diet [7,8,9,10,11,12]. Additionally, the hematopoietic stem cell transplantation causes severe alternations in the developing organism [13,14]. Due to the dysregulation of the blood and gut environment and endothelial toxicities, the organism becomes more sensitive to external factors, including nutrition [15]. Due to severe mucosal toxicities or gastrointestinal complications of GvHD many children required total parenteral nutrition and the return to the normal oral diet is prolonged and difficult [16]. The iatrogenic deprivation of patient homeostasis rises the success rate for the lack of rejection and relapses, but also leads to severe complications [4]. Besides, pediatric HSCT recipients, especially with malignant hematologic disorders, are one of the most abundant groups due to size of the population [17].

At present, there are very few studies that look at the effect of complex oncological treatment including HSCT followed by immunosuppression on lipid profiles of children. These investigations are important because of the potential association of hyperlipidemia and increased risk of future cardiovascular morbidity and mortality in pediatric HSCT recipients requiring early prophylactic and diagnostic approaches. 

The presented study aimed to retrospectively investigate the changes in plasma lipid parameters, thyroid hormone disorders, and time of their onset in pediatric patients after HSCT. 

## 2. Materials and Methods 

### 2.1. Patients Group

The study was performed on the group of 198 patients (mean age 9.33 ± 5.29 years) from the Department and Clinic of Pediatric Oncology, Hematology, and Bone Marrow Transplantation, Wroclaw Medical University. All of the patients (75 females, 123 males) underwent HSCT during the observation period. Most of the patients were diagnosed with Acute Leukemia (190), 5 patients with Chronic Leukemia, and 3 patients with Myelodysplastic Syndromes. 53 donors were relatives of the patients and 145 were unrelated. The origin of the stem cells differed among the patients—163 subjects underwent peripheral blood stem cell transplantation (PBSCT), 34 patients underwent bone marrow transplantation (BMT), and 1 patient underwent cord blood transplantation (CBT). The characteristics of the study group are shown in Table 1.

### 2.2. Data Gathering

**Figure 1:**
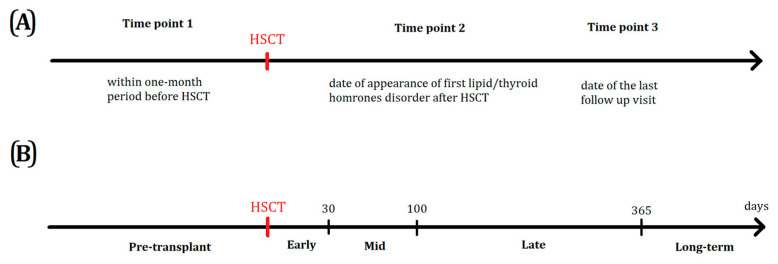
Timelines showing (**A**) the time of lipid and thyroid hormones data gathering due to the expected clinical relevance and (**B**) typical posttransplant periods with respect to graft infusion.

Lipid and thyroid hormone levels were evaluated at three time points: within one month period before HSCT (Time point 1), time of the first lipids/thyroid hormones disorder and symptoms after HSCT—(median time 0.367 ± 1.285 year after HSCT) (Time point 2), and date of last follow up visit in the Department and Clinic of Pediatric Oncology, Hematology and Bone Marrow Transplantation (Time point 3) between 2016 and 2019 (median 3.783 ± 1.809 years after HSCT). Graphic illustration of data gathering time points is shown in Figure 1. 

The analyzed parameters included changes in total cholesterol, LDL-cholesterol (LDL), HDL-cholesterol (HDL), triglycerides (TG) during the therapy. Thyroid metabolism was analyzed by the TSH and free thyroxine (fT4) assessment; anti-thyroglobulin (TgAb), and anti-thyroid peroxidase (TPOAb) antibodies were analyzed as well. Thyroxine treatment was monitored among the patients suffering from hypothyroidism. Body Mass Index (BMI) was estimated based on weight and height measured on last follow up visit (at time point 3). BMI was described in Standard Deviation Score (SDS) to compare the results of patients in different age and sex groups. BMI SDS of the patients varied from −3.72 to 7.31 (mean value 0.50 ± 2.09). Patients were a representative sample due to the mean value of the SDS parameters (formula 1) oscillating around 0 (normal level −1.6−1.6).

(1) $$Standard\;deviation\;score\;\left(SDS\right)=\;\frac{recorded\;value-value\;for\;50th\;centile\;}{\frac{1}{2}\left(value\;for\;50th\;centile-\;value\;for\;3rd\;centile\right)}$$

The privacy of the patients was respected during the analysis by not including their details, such as name and surname. The gathered data was limited to the scientifically valuable clinical data. The personal data used in the analysis included age, gender, and the course of the disease. All subjects gave their informed consent for inclusion before they participated in the study. The study was conducted in accordance with the Declaration of Helsinki. The study was approved by the Ethics Committee of Wroclaw Medical University (No KB-282/2014).

### 2.3. Statistical Analysis

All the statistical analysis of the patients was performed with Statistica 13.3 software (TIBCO, StatSoft, Poland). Depending on the type of the chart, the data was plotted with Statistica or GraphPad Prism 8. U-Mann-Whitney test was incorporated to analyze the differences between LDL, HDL, total cholesterol, triglycerides, TSH, fT4, TPOAb, and TgAb levels among the test group. Spearman correlation tests were performed to investigate the correlations between analyzed lipid factors. Test values under 0.05 (*p* < 0.05) were considered statistically significant. Statistically significant differences between the groups were described and discussed in the following chapters. The analysis that has not revealed any significant data was mentioned only when needed. 

## 3. Results

### 3.1. Lipid Levels Analysis

The group was initially analyzed with respect to the disease type—namely, the patients were classified as ALL and AML. The only statistically significant difference (*p* < 0.003) was the level of cholesterol after HSCT. Patients with ALL were characterized by higher total cholesterol (184 vs 209 mg/dL average; 1.27% vs 1.46% in the age group). That corresponds to steroid therapy prior to transplantation. Due to the fact that this was the only difference inside the AL group, from this point, the patients were analyzed as a whole. 

Generally, the most commonly developed lipid disorder after HSCT was TG level elevation (82.91%), followed by total cholesterol increase (75.00%), and low HDL level (62.96%, Table 2). 

**Figure 2:**
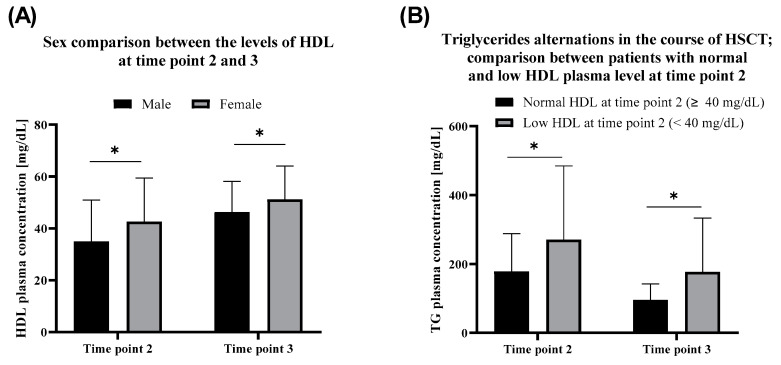
(**A**) Sex comparison between the levels of HDL(high-density lipoprotein) at time point 2 (date of the first appearance of disorder after HSCT) and time point 3 (last follow up visit). (**B**) Triglycerides alternations in the course of HSCT; the comparison between patients with normal and low HDL plasma level at time point 2. *, *p* < 0.01 (U-Mann-Whitney test).

Compared to males, the female patients exhibited relatively higher HDL levels in the first months after the HSCT (time point 2). HDL in the serum of females outreaches the males in the convalescence period after the therapy as well. HDL increase in time after HSCT (time point 3) was observed within both females (*p* = 0.0027) and males (*p* < 0.000001) (Figure 2A). 

Groups with normal and low HDL levels at time point 2 showed no difference in the incidence of TG level disorder before HSCT (*p* > 0.2 at time point 1). Furthermore, since HSCT until time point 3, the average triglycerides level decreased about twofold. The difference between the analyzed groups was observed (TG *p* = 0.000078 at time point 2 and *p* = 0.000044 at time point 3). Namely, during the therapy, TG raised higher in patients with low HDL at time point 2. During the treatment, the level of TG decreased (*p* = 0.000003 for the group with normal HDL at time point 2 and *p* = 0.000409 for the group with low HDL at time point 2) in both groups, although remaining higher in the group with low HDL at time point 2 (Figure 2B). 

**Figure 3:**
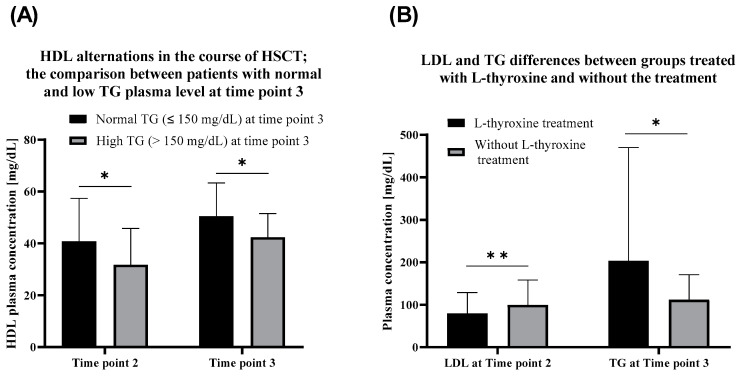
(**A**) HDL alternations in the course of HSCT; the comparison between patients with normal and low triglycerides plasma level (TG) at time point 3. (**B**) LDL (low-density lipoproteins) and triglycerides differences between groups treated with L-thyroxine and the without treatment. *, *p* < 0.01, (U-Mann-Whitney test). **, *p* < 0.05, (U-Mann-Whitney test).

Patients with currently (time point 3) elevated triglycerides exhibited lower HDL at time point 2 as well as at time point 3 than the patients with normal TG levels (Figure 3A). Patients with currently normal TG level exhibited higher HDL serum concentration at time point 2 comparing to the group with currently high TG level. During the observation period, HDL levels tended to increase among both patients with currently high (*p* = 0.000002) and normal (*p* = 0.000086) TG levels. 

TG levels among patients with high and normal LDL before HSCT (at time point 1) did not differ after HSCT (*p* = 0.904, time point 2) and in the recurring time (*p* = 0.932, time point 3). LDL at time point 2 correlated (R = 0.52) with its current level and current cholesterol level (R = 0.17). Total serum cholesterol has not changed during the therapy (*p* > 0.05). No correlation between BMI SDS and lipid levels was observed.

### 3.2. Thyroid Hormones and Thyroid Antibodies Analysis

Fifty-eight patients were affected with thyroid disorders (Table 3), among which 41 were treated with L-thyroxine due to hypothyroidism.

Patients treated with L-thyroxine exhibited lower LDL plasma concentration in comparison to the non-treated group. Conversely, TG is currently on a higher level in patients without thyroxine administration (Figure 3B). TSH levels correlated with total cholesterol at time point 2 (R = 0.22). No correlations between levels of TSH and TG were observed.

**Figure 4:**
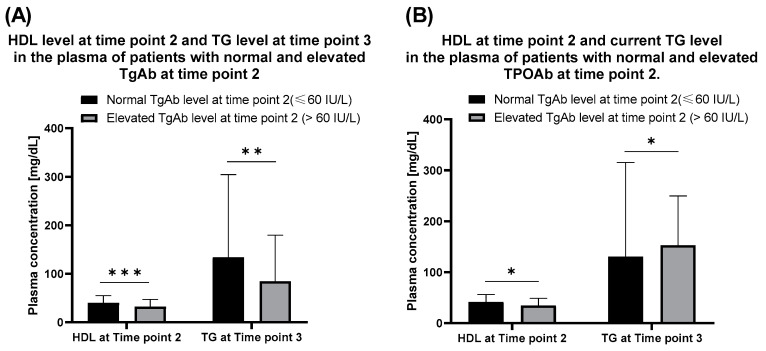
(**A**) HDL at time point 2 (date of the first appearance of disorder after HSCT) and TG level at time point 3 (last follow up visit) in the plasma of patients with normal and elevated TgAb at time point 2. **, *p* < 0.02, (U-Mann-Whitney test). ***, *p* < 0.03, (U-Mann-Whitney test). (**B**) HDL at time point 2 and current TG level in the plasma of patients with normal and elevated TPOAb after HSCT (time point 2). *, *p* < 0.01, (U-Mann-Whitney test).

Thyroid antibodies were evaluated after the HSCT (at time point 2) and served as reference data for further assumptions. The analysis revealed the presence of abnormally high TgAb and TPOAb in 23 and 45 patients, respectively. In some cases, thyroid antibodies did not reveal until a few years after the HSCT procedure. Patients with a normal level of TgAb exhibited increased HDL level at time point 2 (Figure 4A). 

The same was observed with the TPOAb (Figure 4B). Level of TPOAb statistically correlated both with the elevated triglycerides (R = 0.25) and TgAb (R = 0.18). No correlation between thyroid antibodies and time point 3 HDL level was found.

### 3.3. Time Course of Metabolic Complications after Hematopoietic Stem Cell Transplantation (HSCT)

**Figure 5:**
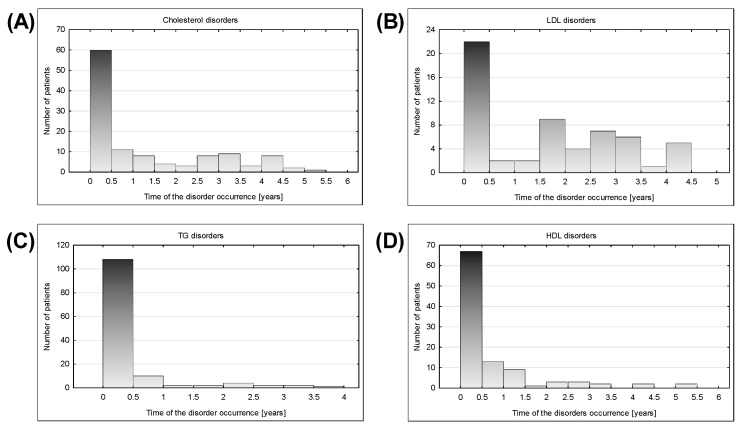
Time distribution of the lipid disorders at time point 2 (disorder first appearance after HSCT) occurrence among patients; x-axis is the time of the disorder occurrence after HSCT [years] and the y-axis is the number of children that developed the disease; (**A**) Cholesterol disorders—Shapiro-Wilk *p* < 0.00001, average 0.726, SD 1.087, variance 1.181, population 113, median 0.275; (**B**) LDL disorders—Shapiro-Wilk *p* < 0.00001, average 1.11, SD 1.363, variance 1.858, population 44, median 0.365; (**C**) Triglycerides disorders—Shapiro-Wilk *p* < 0.00001, average 0.388, SD 0.713, variance 0.509, population 131, median 0.0972; (**D**) HDL disorders—Shapiro-Wilk *p* < 0.00001, average 0.704, SD 1095, variance 1198, population 102, median 0.254.

Disturbances related to LDL, cholesterol, triglycerides, HDL, and the presence of TPOAb occurred mostly in the first half of the year after the HSCT. LDL, HDL, TG, and cholesterol complications occurrence plots (Figure 5) contained only single peak and the occurrences of the thyroid-related complication were more widely distributed in time. 

Lipid complications appeared mostly within 6 months after HSCT, showing a downward trend over time. Disturbances in the first half of the year after HSCT included most often TG level increase (68.35% of tested population), followed by low HDL levels (41.36%), and total cholesterol level elevation (38.46%). Despite the disorders occurring less frequently over time, in the period of 2–4 years after HSCT, there was still a significant group of subjects with newly detected disturbances (Figure 5E).

The exceptional trend in case of LDL level was observed. After decreasing tendency until 1.5 years after HSCT, there was an increased incidence of disorder again (Figure 5).

**Figure 6:**
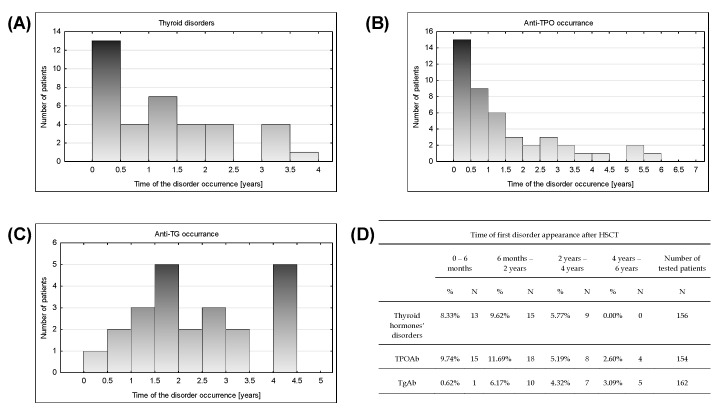
Time distribution of the thyroid disorders at time point 2 (disorder first appearance after HSCT) occurrence among patients; x-axis is the time after HSCT at which the disorder occurred [years] and the y-axis is the number of children that developed the disease; (**A**) Thyroid disorders—Shapiro-Wilk *p* = 0.00299, average 1.241, SD 1.06, variance 1.123, population 37, median 1.011; (**B**) Anti-TPO antibodies occurrence after HSCT—Shapiro Wilk *p* = 0.00001, average 1454, SD 1514, variance 2292, population 45, median 0.981; (**C**) Anti-TG antibodies occurrence after HSCT—Shapiro Wilk *p* = 0.317, average 2.371, SD 1.219, variance 1486, population 23, median 2217; (**D**) Table with the data used for the analysis.

In the presence of thyroid antibodies and thyroid disorders, metabolic complications had two peaks in time (Figure 6). Within six months after HSCT, most of the thyroid hormone complications and increased levels of TPOAb were detected (Figure 6). Thyroid hormone abnormalities were evenly distributed in time until 4 years after HSCT without new occurrences later. TPOAb revelation tended to decrease in time but was still observed in the period of 4–6 years after HSCT (Figure 6B).

TgAb elevation appearance showed an upward trend until 1.5 years after HSCT (Figure 6C). After even detection until 3.5 years, a leap of occurrence of TgAb increase in period 4–4.5 year was noticed. 

**Figure 7:**
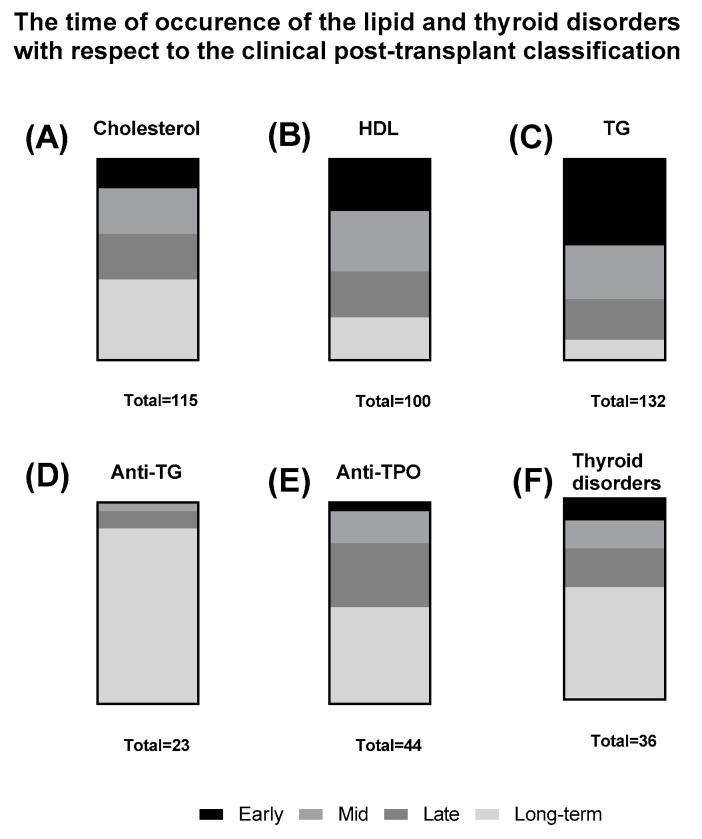
The time of lipid and thyroid disorders occurrence with respect to the clinical post-transplant classification. The boxes represent the percentage of each class among patients that had the specific lipid disorder. (**A**) cholesterol disorder (*n* = 115); (**B**) HDL disorder (*n* = 100); (**C**) Triglycerides disorder (*n* = 132); (**D**) Anti-TG occurrence (*n* = 23); (**E**) Anti-TPO occurrence (*n* = 44); (**F**) Thyroid disorder (*n* = 36).

Interestingly, when the data concerning the occurrence of the lipid and thyroid disorders was grouped with respect to the clinical classification, high distinction between the lipid disorders was observed. Total cholesterol disorders were observed mostly in the long-term follow up (Figure 7A), HDL disorders were more evenly distributed between the classes (Figure 7B) and triglycerides disorders occurred mostly in the early period of the follow up (Figure 7C). Conversely, the thyroid-associated disorders and anti-thyroid antibodies were most prevalent in the long-term follow up of the patients (Figure 7D–F). 

## 4. Discussion

Although the alternations in blood proteome have already been extensively described, the lipid alternations in the course of HSCT remain unclear [14,18]. The analysis of our patients presented in this paper revealed some regularities in the lipid metabolism during the course of HSCT. First, our analysis revealed the lack of changes in cholesterol and cholesterol blood carrier proteins—LDL during the course of HSCT. The levels of HDL were not affected by the transplantation as well. Literature reports that the production of the ApoB-100 is associated with liver [19,20]. Thus, the HSCT procedure does not affect the production rate of the LDL associated proteins. More interestingly, our study reports that lipid metabolism were not affected as well, namely HSCT did not exert any effect on the lipid biosynthesis. The transplant procedure has not affected the tendency for higher HDL plasma levels in female over male patients, which stays in agreement with the widely described phenomenon, that females exhibit higher HDL-plasma levels [21]. Hepatic lipase in the liver-associated enzyme is responsible for the differences in HDL content between both genders [22]. The hydrolytic enzyme’s expression remains under the control of estrogens [23,24]. Therefore, in female patients, the conversion of HDL3 to HDL2 is elevated [23].

This study revealed that the only lipid-associated factor affected by the HSCT is the HDL and triglycerides cooperative regulation. Namely, the progression in triglycerides levels after the HSCT occurred among patients leading to the currently low-HDL plasma content. Patients with currently elevated triglycerides exhibited high HDL alongside the triglycerides after the HSCT. The relatively opposite regulation of low-in-triglycerides HDLs and full-of-triglycerides low-density lipoproteins (VLDL, LDL, and chylomicrons) explains the observed phenomenon [25]. However, standard deviation (SD) used for descriptive error bars in the statistical analysis section are quite high, especially for TG levels. The presented data can be spread due to the sample size of the chosen population, oncological pediatric patients undergoing HSCT, and sensitivity of the parameter by itself that biased final clear interpretation. A mentioned issue revealed how significant it is to inform patients about keeping a balanced diet, as well as fasting requirements on the day preceding the laboratory tests of lipid profile. Authors’ own experiences show that the nighttime meals and diet, including fast-food on the day preceding blood sampling, cause remarkable disturbances in level of triglycerides. Deviations from a healthy diet can cause transient lipid disorders, but constant poor diet leads to persistent bad eating habits, constant disorders, and metabolic syndrome. Patients undergoing chemotherapy should pay particular attention to healthy dietary patterns with low fat intake.

Changes observed in lipid metabolism could be a result of the hormonal level imbalance [26]. Mostly, thyroid is responsible for the energetic state of the organism [27]. Therefore, the complications with the hormones secreting organ affect the fatty tissue and steroidogenic tissues. Due to the fact that lipid metabolism remains the most effective energy-supplying source for the organism, even slight alternations in thyroid hormones levels severely affect the plasma lipoproteins [26,28,29,30]. The patients who supplied the thyroid hormones exhibited lower LDL and higher triglycerides levels comparing to the non-treated group. The occurrence of TPOAb and TgAb correlated with the decrease of the thyroid hormones secreting function. Follow-ups, including thyroid function testing with thyroid antibodies assessment, should cover a period of at least 4 years after HSCT. Therefore, the patients with the elevated auto-antibodies were characterized by the increase in low-in-lipids HDL and decrease in plasma triglycerides.

The vast majority of the lipid-associated complications occurred in the first half of the year after HSCT. The changes in triglyceride level may be the result of CSA and glucocorticoids used in the immunosuppression process. The increased fatty acids biosynthesis leads to an increase in the triglyceride’s plasma levels. Due to the fact that triglycerides are mostly transported by VLDL, and not by LDL or HDL, the concentrations of the others remain constant in the course of the transplantation [31,32]. The lack of late (over 6 months) lipid complications could almost arise from the high proliferation rate and the ease of adaptation into the new microenvironment of the transplanted bone marrow [33].

Because of the frequently occurring lipid disorders within six months after HSCT, pediatric patients undergoing transplantation procedure should follow a diet with saturated fat restrictions, rich in foods containing unsaturated fatty acids. Introduction of polyunsaturated fat to the diet with simultaneous limiting of saturated fatty acids reduce TG and LDL levels due to decrease LDL production rates with elevating LDL clearance rates [34]. Application of the mentioned dietary recommendations should also cause HDL level reduction. However lowering of LDL level is much more significant and a clinically beneficial elevation of HDL/LDL ratio is observed [35]. The change in diet is especially important for males, who are more likely to develop adverse lipid disorders. Patients treated with L-thyroxine require not only laboratory assessment of thyroid function, but also introducing healthy diets with lipid levels evaluation due to the higher risk of developing lipid disorders.

## 5. Conclusions

Various lipid complications occur after HSCT in pediatric patients. Therefore, clinicians should include the lipidomic studies in the follow-ups after the therapy. Our results show that pediatric patients after HSCT exhibit most of the lipid-related complications in the first half of the year after the therapy. There is no gender specificity in the occurrence of lipid disorders caused by HSCT. The most common lipid disorders after HSCT were increased levels of TG and total cholesterol and decrease of HDL level. Curiously, during the therapy, TG raised higher in patients with low HDL after HSCT. Normal TG level implicated higher HDL serum concentration after HSCT and after treatment. Hypothyroidism patients on thyroid hormones therapy exhibited lower LDL and higher TG plasma concentration in comparison to the non-treated group. Introduction of healthful dietary pattern including replacement saturated fat with polyunsaturated fatty acids can reduce risk of adverse lipid disorders in patients after HSCT. It is needed to conduct further international, multicenter studies on lipid metabolism and thyroid gland function in posttransplant cancer survivors, especially due to higher and higher effectiveness of treatment and longer survival time of patients.

## Figures and Tables

**Table 1:** Characteristics of the examination group.

Parameter	Value
Number of patients	198
Age (y)	0.5–25
Mean age (y)	9.33 ± 529
Gender	*N (%)*
Male	123 (62.12%)
Female	75 (37.88%)
Diagnosis	*N (%)*
Acute Leukemia	190 (95.96%)
Acute Lymphoblastic Leukemia	119 (60.10%)
Acute Myeloid Leukemia	71 (35.86%)
Chronic Myeloid Leukemia	5 (2.53%)
Myelodysplastic Syndrome	3 (1.51%)
HSC source	*N (%)*
Peripheral blood	163 (82.32%)
Bone marrow	34 (17.17%)
Cord blood	1 (0.51%)
Donor type	*N (%)*
Unrelated	145 (72.68%)
Related	53 (27.32%)

**Table 2:** Frequency of developing lipid disorders after hematopoietic stem cell transplantation (HSCT) in the analyzed group of patients.

	Number of Patients with the Disorder		Total Number of Tested Patients
	%	*N*	*N*
Total cholesterol	75.00%	117	156
LDL	37.66%	58	154
HDL	62.96%	102	162
TG	82.91%	131	158

**Table 3:** Frequency of developing thyroid function disorders after HSCT in the analyzed group.

	Number of Patients with the Disorder		Total Number of Tested Patients
	%	*N*	*N*
Thyroid hormones’ disorders	37.66%	58	154
TPOAb	33.83%	45	133
TgAb	17.29%	23	133
